# Chronic kidney disease of unknown aetiology: a real-world study

**DOI:** 10.1186/s12882-025-04452-1

**Published:** 2025-10-24

**Authors:** Joshua Storrar, Sayyid Raza, Ivona Baricevic-Jones, Sharmilee Rengarajan, Rajkumar Chinnadurai, Philip A. Kalra, Smeeta Sinha

**Affiliations:** 1 Donal O’Donoghue Renal Research Centre, Northern Care Alliance NHS Foundation Trust, Salford Royal Hospital, Stott Lane, Salford, M6 8HD UK; 2 Faculty of Biology, Medicine and Health, University of Manchester, Oxford Road, Manchester, UK

**Keywords:** Chronic kidney disease, Cohort study, Epidemiology, Rare kidney disease, Unknown aetiology

## Abstract

**Background:**

Diagnosis of rare chronic kidney disease (CKD) can be difficult with conventional diagnostic workup. When no diagnosis is identified, this is termed CKD of unknown aetiology (CKDUA). There are multiple benefits in obtaining a diagnosis in these cases. The advent and increasing availability of genetic testing in recent years has been a welcome additional diagnostic tool. In this study we aimed to determine the demographics and kidney related outcomes for a cohort of patients with CKDUA within the Salford Kidney Study (SKS).

**Methods:**

The SKS is a single-centre, ongoing, prospective, observational cohort study of adult patients referred to the renal service at Salford Royal Hospital, UK. Within the SKS there are 398 patients with CKDUA. A group with diabetic kidney disease (DKD) was used as a comparator. An analysis was performed comparing these two groups with a particular focus on their demographics and kidney related outcomes.

**Results:**

Median age of the CKDUA cohort was 71.4 years, with 60.1% male and 96.2% White. There was advanced CKD at presentation (median eGFR 30 ml/min/1.73m^2^), with a 5-year mortality of 27.6%. When compared with the DKD cohort, the CKDUA cohort were older (71.4 years vs. 67.3 years, *p* < 0.001), had more equal sex distribution (male 60.1% vs. 68%, *p* < 0.009), had less advanced renal disease at presentation (eGFR 30 ml/min/1.73m^2^ vs. 26 ml/min/1.73m^2^, *p* < 0.001), and were less likely to progress to renal replacement therapy (14.1% vs. 28.2%, *p* < 0.001).

**Conclusions:**

CKDUA is difficult to manage: other than general supportive measures there is little else that can be offered. This study reports on a real-world cohort of patients with CKDUA and identifies the surprising finding that in comparison to a DKD cohort their outcomes are improved, with a significantly lower rate of progression to RRT.

**Clinical trial number:**

Not applicable.

**Supplementary Information:**

The online version contains supplementary material available at 10.1186/s12882-025-04452-1.

## Background

Diagnosis of rare chronic kidney diseases (CKD) can be difficult with conventional diagnostic workup (laboratory tests including standard blood and urine investigations, imaging, and kidney biopsy). As such, a proportion of patients with rare CKD do not receive an underlying diagnosis. Their CKD is labelled CKD of Unknown Aetiology (CKDUA). CKDUA represents about 10% of patients diagnosed with end-stage kidney disease (ESKD) [1].

There are several benefits to receiving a diagnosis and these include: (1) potential change in management with the use of specific treatment (for example enzyme replacement therapy in Fabry’s disease), (2) helping to guide kidney transplantation planning, (3) helping to screen potentially affected family members, (4) assisting with reproductive options, (5) highlighting possible extra renal features/renal sub-diseases which can potentially be screened for and detected earlier, (6) avoiding ineffective therapies in some cases, (7) guiding clinicians and patients regarding clinical trials in a specific disease area and (8) preventing progression towards ESKD and the need for costly renal replacement therapy (RRT) [2].

It has been shown that the diagnostic yield for patients with CKDUA following genetic testing, and targeted testing for specific conditions such as Fabry’s disease, is between 12 and 56% [2–8], with whole exome sequencing (WES) performing better than targeted gene panels. At our centre we have a large prospective observational study known as the Salford Kidney Study (SKS) with currently over 3,500 patients enrolled since 2002. Within it, there are over 400 patients (11%) with CKDUA [9]. Extrapolated to our whole outpatient population this figure increases to around 1100–1650 patients. This suggests that there are a large number of patients within our care without a known cause to explain their CKD. A proportion of these are likely to have an underlying genetic diagnosis, including a variety of monogenic causes encompassing the whole range of kidney conditions (for example autosomal dominant polycystic kidney disease [ADPKD], glomerulopathy related to collagen type 4 alpha 1 chain [COL4A] mutations, and autosomal dominant tubulointerstitial disease [ADTKD] caused by uromodulin [UMOD] or mucin-1 [MUC-1] mutations). Whilst some of these diagnoses will already be apparent prior to genetic testing (most patients with ADPKD are diagnosed based on ultrasound scan findings and the presence of a family history), it has been shown that when WES was performed on a large cohort of patients with kidney disease, the majority of the diagnoses that were revealed each occurred in one patient only [1], highlighting the rarity of these conditions which are unlikely to be diagnosed prior to testing.

There have been no previous studies comparing clinical outcomes of CKDUA with common causes for CKD, perhaps reflecting the lack of an agreed consensus on what constitutes CKDUA. However, this is starting to change with the recently published consensus statement from the Genes and Kidney Working Group of the European Renal Association which aims to standardise how CKD of unexplained cause is recorded and the nomenclature that should be used [10].

Historically, CKDUA patients have been poorly served: it attracts less research interest, treatment options are limited and there is often a lack of awareness of rare kidney disease. This has changed over more recent years with the increasing availability of genetic testing, but this comes with its challenges including the need to upskill nephrologists and to develop strong links with geneticists. There are several aims of this study:

1. To discover demographic characteristics of patients with CKDUA (within the Salford Kidney Study [SKS]);
2. To determine their kidney-related outcomes;
3. To determine what phenotypic or laboratory features will help diagnose these patients earlier on in their pathway; and.
4. To determine the risk factors that increase the likelihood of patients having CKDUA.

Understanding the above may allow us to develop clinical algorithms and a subsequent stratification plan, allowing patients to be identified and targeted earlier for genetic testing.

## Methods

### Study population

The SKS is a single-centre, ongoing, prospective, observational cohort study of adult patients referred to the renal service at Salford Royal Hospital, Northern Care Alliance NHS Foundation Trust, UK. Patient recruitment initially commenced in 2002 and the current database has greater than 3500 patients, making it one of the largest international epidemiological studies involved in all aspects of CKD care and research. The renal service at Salford Royal Hospital serves a direct population of approximately 240,000 people in the City of Salford and this is included in the overall 1.55 million catchment population in the surrounding North West region of the UK, which includes the local authorities of Bolton, Bury, Oldham, Rochdale and Wigan.

The inclusion criteria into SKS include:

- Age ≥ 18 years at the time of consent.
- Referred to or under the care of renal services at Salford Royal Hospital (see NICE guideline NG203 for referral criteria) [11].
- eGFR < 60 ml/min/1.73m^2^ in the preceding 12 months.
- Able to give written informed consent for participation.

Exclusion criteria include:

- Age < 18 years at time of consent.
- Not known to have CKD or had an episode of AKI with eGFR < 60 ml/min/1.73m^2^.
- Unable to give written informed consent for participation.

Patients suitable for enrolment are provided with a patient information sheet and if they are willing to participate, written informed consent is obtained at the next clinic visit by a trained research nurse. All patients enrolled into the SKS give full written consent for their data to be used for research puprposes.

**Fig. 1:**
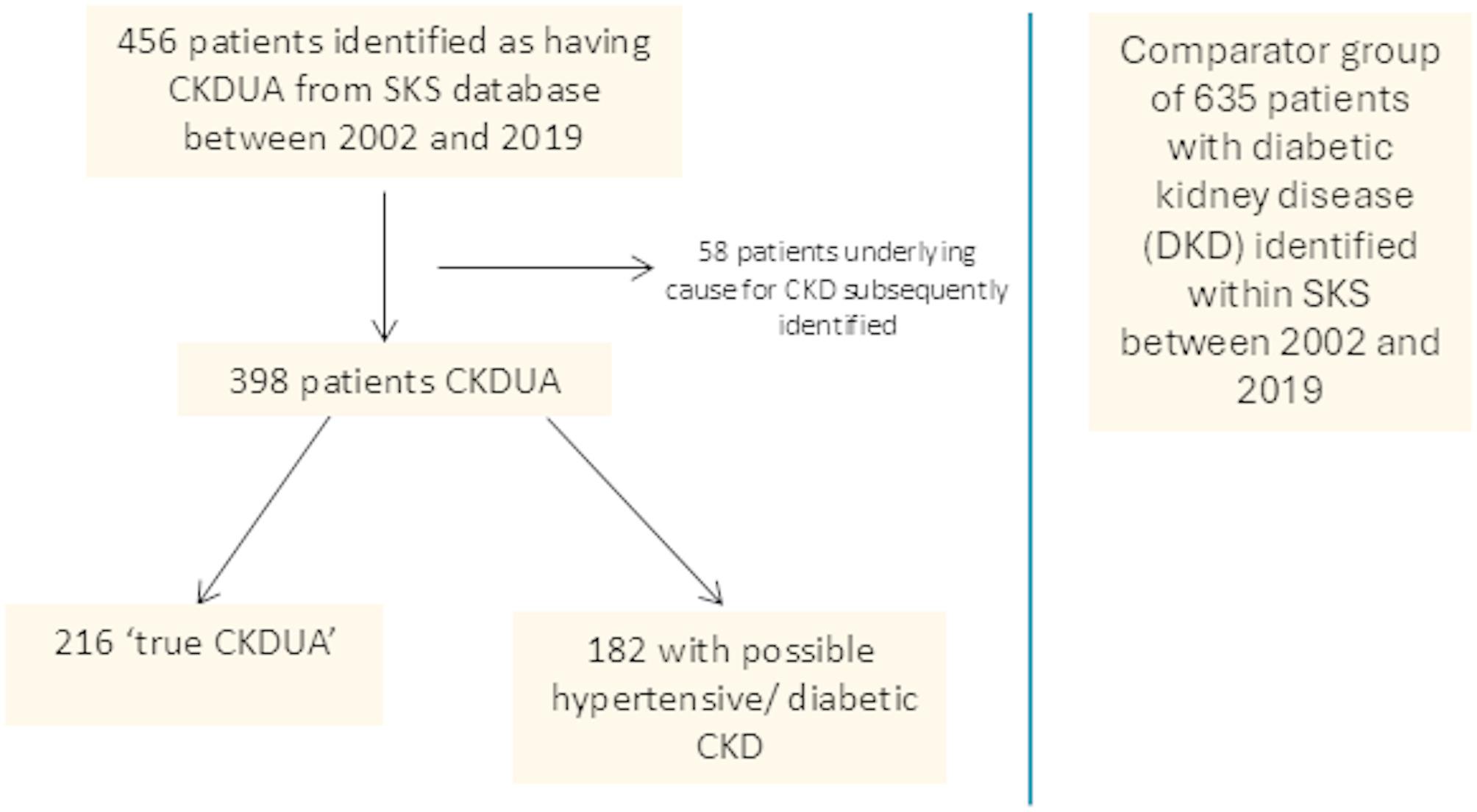
Flowchart of patient involvement in the study

Patients with a diagnosis code of CKDUA were identified from the SKS database; this initially totaled 456 patients. On subsequent review, 58 did have an underlying cause for their CKD identified. These included reno-vascular disease, long-term lithium use, episodes of acute kidney injury, radical nephrectomy and cardio-renal disease. This resulted in 398 patients in the total cohort. Figure 1 shows the flowchart of patient recruitment to the study. The cohort was further split into those with ‘true CKDUA’ and a group who were found to have possible underlying hypertensive or diabetic kidney disease (H/D CKD) based on a review of their electronic patient record including clinic letters from nephrology outpatient clinics. The underlying hypertension and diabetes had not been directly attributed to their CKD but was possibly a factor in its development. Nephrologists were instrumental in identifying potential underlying aetiology through a combination of assessing clinical information and reviewing biopsy results where appropriate.

A wide variety of clinical data is obtained at recruitment and updated on an annual basis in the SKS by dedicated research nurses and trained clinical research fellows using detailed questionnaires and reference to clinical care records where needed. All aspects of data collection are performed at patients’ routine clinic visits to avoid the need for repeated attendances. Data collected falls under the following categories: demographics, primary renal disease, comorbidities, medications, blood pressure and laboratory data. Estimated GFR values are calculated using the CKD-EPI_2021_ equation.

For this study, additional information relating to specific features that might give a clue to the underlying cause of CKD was also collected from the electronic patient record (EPR) (including complement levels, family history of kidney disease, connective tissue disease screen abnormality, kidney size, cysts seen on imaging).

A group of patients within SKS with diabetic kidney disease (DKD) (*n* = 635) was used as a comparator group. DKD was defined as either those with biopsy confirmed diabetic nephropathy; or in those who had not undergone kidney biopsy then CKD co-existing with other diabetic complications without an alternative explanation for their CKD; or in those where clinically diabetic nephropathy was felt to be the underlying cause for their CKD. The rationale for using DKD as the comparator group is that it is a well defined condition (not a group of heterogeneous disorders such as glomerulonephropathies), with patients on average not progressing to ESKD quickly. Kidney biopsy results for those patients who underwent a biopsy were obtained from the EPR.

### Ethical considerations

SKS has been granted approval from the North West Greater Manchester South Research Ethics Committee (REC15/NW/0818). This study was conducted in accordance with the declaration of Helsinki.

### Statistical analysis

SPSS (IBM) version 25 licensed to the University of Manchester was used for statistical analysis. Analysis of the original overall cohort was undertaken and compared with the DKD cohort. A comparison was made between baseline demographics, comorbidities, laboratory values and outcomes including progression to renal replacement therapy (RRT) and mortality. A similar comparative analysis was made between the true CKDUA group and the H/D CKD group. In the descriptive part of the analysis continuous variables are presented as median (interquartile range) and the Mann- Whitney U-test is used to test statistical significance. Categorical data are expressed as number (percentage), and the Chi-square test is used for statistical significance. A p-value of < 0.05 was taken as statistical significance throughout the analysis.

Urine protein creatine ratio (uPCR) values are presented in mg/mmol. To convert this to a value in g/g then multiply by 0.113. Conversely, to convert from g/g to mg/mmol, multiply by 8.84. Creatinine values are presented in µmol/l. To convert this to a value in mg/dl then multiply by 0.0113.

Delta eGFR (annual rate of change of eGFR) was calculated by linear regression analysis using all available eGFRs between the study baseline and endpoints. Only patients with three or more eGFRs were included in this analysis.

The association of baseline variables with a requirement for RRT and mortality was calculated using univariate and multivariate Cox proportional hazard models to determine hazard ratios (HRs), 95% confidence intervals (CIs) and statistical significance.

## Results

The baseline characteristics, laboratory results and outcomes for the CKDUA cohort are shown in Table 1. The median age was 71.4 years (interquartile range [IQR] 61.9–78.8), 60.1% were male and 96.2% were White. The median body mass index (BMI) was 28.3 kg/m^2^ (IQR 24.4–32.5). CKD was advanced at presentation with median creatinine 173µmol/L (IQR 132–249) and eGFR 30 ml/min/1.73m^2^ (IQR 20–44). Progression to RRT was seen in 14.1%, mortality was observed in 55.3%, 5-year mortality was seen in 27.6%, and median follow-up duration was 44 months (IQR 21–82).

**Table 1:** Baseline characteristics, blood results and outcomes for both CKDUA and DKD

Variable	CKDUA (398)	DKD (635)	*P*-value
Age, years	71.4 (61.9–78.8)	67.3 (58.9–74.5)	**< 0.001**
Male	239 (60.1)	432 (68.0)	**0.009**
White	383 (96.2)	603 (95.0)	0.34
BMI, kg/m^2^	28.3 (24.4–32.5)	30.3 (26.6–35.1)	**< 0.001**
SBP, mmHg	140 (125–152)	142 (128–160)	**0.020**
DBP, mmHg	73 (66–80)	72 (62–80)	0.059
Hypertension	341 (85.7)	612 (96.4)	**< 0.001**
Diabetes	65 (16.3)	623 (98.1)	**< 0.001**
Angina	74 (18.6)	169 (26.6)	**0.003**
MI	51 (12.8)	121 (19.1)	**0.009**
CCF	67 (16.8)	147 (23.1)	**0.015**
CVA	25 (6.3)	68 (10.7)	**0.016**
PVD	47 (11.8)	107 (16.9)	**0.027**
Cancer	57 (14.3)	48 (7.6)	**< 0.001**
Liver disease	9 (2.3)	19 (3.0)	0.481
COPD	77 (19.3)	111 (17.5)	0.449
ACEi/ARB	199 (53.1)	434 (72.9)	**< 0.001**
Statin	196 (52.3)	455 (76.5)	**< 0.001**
Creatinine, µmol/L	173 (132–249)	205 (159–280)	**< 0.001**
eGFR, ml/min/1.73m^2^	30 (20–44)	26 (18–35)	**< 0.001**
uPCR, g/mol	19.5 (10.0-57.7)	65.1 (21.0-216)	**< 0.001**
Albumin, g/L	43 (41–45)	42 (39–44)	**< 0.001**
Hb, g/L	122 (112–133)	117 (108–128)	**< 0.001**
Calcium, mmol/L	2.31 (2.21–2.39)	2.30 (2.21–2.40)	0.634
Phosphate, mmol/L	1.10 (0.98–1.27)	1.20 (1.04–1.38)	**< 0.001**
RRT	56 (14.1)	179 (28.2)	**< 0.001**
5 year RRT rate	46 (11.6)	143 (22.5)	**< 0.001**
Mortality	220 (55.3)	381 (60.0)	0.134
5 year mortality rate	110 (27.6)	211 (33.2)	0.059
Follow up duration, months	44 (21–82)	39 (20–74)	0.235

ACEi, angiotensin converting enzyme inhibitor; ARB, angiotensin receptor blocker; BMI, body mass index; CCF, congestive cardiac failure; COPD, chronic obstructive pulmonary disease; CVA, cerebrovascular accident; DBP, diastolic blood pressure; eGFR, estimated glomerular filtration rate; Hb, haemoglobin; MI, myocardial infarction; PVD, peripheral vascular disease; RRT, renal replacement therapy; SBP, systolic blood pressure

Categorical values presented as number (percentage), continuous variables as median (interquartile range)

The baseline demographic characteristics, laboratory results and the outcome data were compared between the CKDUA and the DKD cohorts (results shown in Table 1). The patients in the CKDUA cohort were older in comparison to the DKD cohort − 71.4 years vs. 67.3 years (*p* < 0.001), they had more gender parity (male 60.1% vs. 68%, *p* < 0.009), had a lower BMI (28.3 kg/m^2^ vs. 30.3 kg/m^2^, *p* < 0.001), lower rate of hypertension and diabetes (85.7% vs. 96.7%, *p* < 0.001 and 16.3% vs. 98.1%, *p* < 0.001, respectively), were less likely to be receiving an angiotensin converting enzyme inhibitor (ACEi)/ angiotensin receptor blocker (ARB) or statin (53.1% vs. 72.9%, *p* < 0.001 and 52.3% and 76.5%, *p* < 0.001, respectively), had less advanced renal disease at presentation (eGFR 30 ml/min/1.73m^2^ vs. 26 ml/min/1.73m^2^,*p* < 0.001), had lower proteinuria (uPCR 19.5 g/mol vs. 65.1 g/mol, *p* < 0.001), were less likely to progress to RRT (14.1% vs. 28.2%, *p* < 0.001) and had lower mortality (although this did not reach statistical significance: 55.3% vs. 60%, *p* = 0.134).

Data on specific clinical, laboratory or other relevant features that might point towards a possible underlying cause for the CKDUA are also recorded for the CKDUA cohort (Table 2). C3 complement levels were outside of the normal range in 9.6% of those who had a result available, and C4 complement levels were outside of the normal range in 2.4% of those who had a result available. A connective disease screen abnormality was observed in 25.3% of patients who had been tested, serum electrophoresis was abnormal in 8.8% of patients who had been tested, a positive family history for kidney disease was recorded in just 1% of patients, renal cysts were seen on imaging in 39.1% of patients of patients who had undergone imaging, and kidney sizes were normal bilaterally (median right kidney 9.8 cm and left kidney 9.9 cm).

**Table 2:** Specific clinical, laboratory or other features of the CKDUA cohort

Variable	CKDUA (total *n* = 398)
C3 outside of normal range (*n* = 250)	24 (9.6)
C4 outside of normal range (*n* = 250)	6 (2.4)
Connective tissue disease screen abnormality (*n* = 300)	76 (25.3)
Serum electrophoresis Abnormality (*n* = 283)	25 (8.8)
Positive family history for kidney disease (*n* = 398)	4 (1.0)
Renal cysts identified on imaging (*n* = 286)	112 (39.1)
Right kidney size, cm	9.8 (8.8–10.5)
Left kidney size, cm	9.9 (9.0-10.7)
Discrepancy in kidney size > 3 cm (*n* = 260)	10 (3.8)

Categorical values presented as number (percentage), continuous variables as median (interquartile range)

There were no specific factors associated with progression to RRT or mortality (beyond standard risk factors) (see supplemental material including Cox regression analysis).

It was identified that a large proportion of CKDUA patients had underlying hypertension and diabetes which was speculated to be a causative factor for their CKD. This data was obtained by a single author, JS, searching through individual patient clinic letters in the EPR, and making a clinical judgement about the likely underlying contribution to the participants CKD. To explore whether this would impact on the analysis, the total CKDUA cohort was split into two further groups: those with ‘true CKDUA’ (*n* = 216), and those with hypertension and diabetes as possible aetiologic causes ‘H/D CKD’ (*n* = 182). The main differences when comparing the ‘true CKDUA’ with the ‘H/D CKD’ were a lower BMI (median 26.3 kg/m^2^ vs. 29.2 kg/m^2^, *p* < 0.001), less use of ACEi/ARB and statins (40.4% vs. 67.2% and 47% vs. 58.2% respectively), and unsurprisingly lower rates of hypertension and diabetes (Table 3). There was a similar rate of eGFR decline per year between the 2 groups: -0.72 ml/min/1.73m^2^/year (-2.65 to 1.01) vs. -0.76 ml/min/1.73m^2^/year (-2.1 to 1.10).

**Table 3:** CKDUA cohort split according to possible underlying aetiology

Variable	True CKDUA (216)	CKD of possible hypertensive/ diabetic aetiology (H/D CKD) (182)	*P*-value
Age, years	71.3 (61.8–77.8)	72.0 (63.2–79.3)	0.267
Male	130 (60.2)	109 (59.9)	0.952
White	208 (96.3)	175 (96.2)	0.941
BMI, kg/m^2^	26.3 (23.2–31.6)	29.2 (26.0-33.9)	**< 0.001**
SBP, mmHg	139.5 (125–150)	140 (125-156.5)	0.207
DBP, mmHg	75 (68–80)	70 (65–80)	0.215
Hypertension	166 (76.9)	175 (96.2)	**< 0.001**
Diabetes	18 (8.3)	47 (25.8)	**< 0.001**
Angina	46 (21.3)	28 (15.4)	0.131
MI	30 (13.9)	21 (11.5)	0.485
CCF	35 (16.2)	32 (17.6)	0.714
CVA	12 (5.6)	13 (7.1)	0.516
PVD	22 (10.2)	25 (13.7)	0.274
Cancer	34 (15.7)	23 (12.6)	0.379
Liver disease	3 (1.4)	6 (3.3)	0.202
COPD	41 (19.0)	36 (19.8)	0.841
ACEi/ARB	80 (40.4)	119 (67.2)	**< 0.001**
Statin	93 (47)	103 (58.2)	**0.03**
Creatinine, µmol/L	173 (129–256)	171 (132–236)	0.879
eGFR, ml/min/1.73m^2^	31 (20–43)	30 (21–45)	0.998
Annual rate of change of eGFR, ml/min/1.73m^2^/year	-0.72 (-2.65 to 1.01)	− 0.76 (-2.1 to 1.10)	0.641
uPCR, g/mol	18.6 (10.2–56.4)	20.8 (10.4–66.3)	0.586
Albumin, g/L	43 (41–45)	43 (41–45)	0.265
Hb, g/L	123 (111–132)	121 (112–134)	0.410
Calcium, mmol/L	2.32 (2.22–2.41)	2.28 (2.19–2.37)	**0.019**
Phosphate, mmol/L	1.10 (0.98–1.27)	1.10 (0.96–1.26)	0.482
C3, g/L	1.18 (0.95–1.37)	1.22 (1.09–1.42)	**0.044**
C4, g/L	0.25 (0.20–0.31)	0.25 (0.20–0.31)	0.865
CTD screen abnormality	35 (21.5)	41 (29.9)	0.094
Serum electrophoresis abnormality	13 (6.0)	12 (6.6)	0.754
Positive family history	4 (1.9)	0 (0)	0.065
Renal cysts on imaging	57 (36.8)	55 (42)	0.368
Right kidney size, cm	9.6 (8.8–10.5)	9.9 (9.0-10.5)	0.328
Left kidney size, cm	9.6 (8.7–10.5)	10.0 (9.3–10.9)	**0.026**
RRT	31 (14.4)	25 (13.7)	0.846
5 year RRT rate	25 (11.6)	21v(11.5)	0.991
Mortality	109 (50.5)	111 (61)	**0.035**
5 year mortality rate	58 (26.9)	69 (37.9)	**0.018**
Follow up duration, months	47 (22–83)	43 (20–79)	0.719

ACEi, angiotensin converting enzyme inhibitor; ARB, angiotensin receptor blocker; BMI, body mass index; CCF, congestive cardiac failure; COPD, chronic obstructive pulmonary disease; CTD, connective tissue disease screen; CVA, cerebrovascular accident; DBP, diastolic blood pressure; eGFR, estimated glomerular filtration rate; Hb, haemoglobin; MI, myocardial infarction; PVD, peripheral vascular disease; RRT, renal replacement therapy; SBP, systolic blood pressure

Categorical values presented as number (percentage), continuous variables as median (interquartile ran

There was no difference in progression to RRT or 5 year RRT rate, but there was improved overall mortality in the ‘true CKDUA’ group (50.5% vs. 61%, *p* = 0.035), as well as 5 year mortality rate (26.9% vs. 37.9%, *p* = 0.018).

Finally, we looked at the kidney biopsy results in patients who had a biopsy performed within the CKDUA cohort (*n* = 40, 10.1%), to see if there were any features on biopsy that would suggest an underlying aetiology. Table 4 details the specific biopsy features. The most common findings were no obvious histological abnormality (*n* = 9), tubulointerstitial changes (*n* = 9) and advanced chronic damage (*n* = 9). One biopsy identified features of chronic pyelonephritis to suggest an underlying diagnosis. Whilst kidney biopsy was not helpful in making a diagnosis in these cases, it is important to note that many patients not included in this cohort (and thus will by definition have had an underlying cause for their kidney disease identified) are likely to have had a biopsy that helped to make a diagnosis.

**Table 4:** Specific features seen on kidney biopsy in those where it was performed

	Frequency (*n* = 40, 10.1%)
No obvious histological abnormality	9
Tubulointerstitial changes	9
Advanced chronic damage	9
Non-specific appearances. No cause for renal impairment apparent.	5
Moderate to marked chronic damage	2
Mild chronic damage	1
Wrinkling of basement membranes	1
Mesangial matrix increase with some sclerosis and tuft adhesion	1
Subscapular scarring and large number of globally sclerosed glomeruli	1
Periglomerulus sclerosis with some sclerosed glomeruli. Chronic inflammation within the interstitium. Inflammatory cell infiltrate: lymphocytes, plasma cells and eosinophils. Appearances are those of chronic pyelonephritis.	1
Several biopsies: difficult to interpret due to multiple features	1
Biopsy inadequate for assessment	1
Mild to moderate interstitial fibrosis and tubular atrophy	1

## Discussion

The aim of this study was to investigate a real-world cohort of unselected patients labelled with CKDUA. Our main findings were that: (1) patients were at a relatively older age at the start of study enrolment; (2) they had advanced kidney disease (median eGFR 30 ml/min/1.73m^2^); (3) there was less progression to RRT compared to the DKD cohort (14.1% vs. 28.2%); (4) mortality was better in the ‘true CKDUA’ group compared to the hypertensive/ diabetic phenotype “H/D CKD” (50.5% vs. 61%, *p* = 0.035); (5) a positive family history was noted in only 1% of cases; and (6) there were no specific factors associated with progression to RRT/ mortality in addition to standard risk factors associated with more advanced CKD.

It is well established that CKDUA is difficult to manage. Other than general supportive measures there is no specific treatment that can be offered for these patients. However, it is known that a proportion of patients will have an underlying genetic cause, and a number of papers published recently considered genetic diagnoses in cohorts with both CKD and CKDUA [1, 7, 12–14].

A seminal paper by Groopman et al. [1]. reported on WES performed in over 3000 patients with CKD, with a diagnostic yield of 9.3% involving 66 different monogenic disorders. The diagnostic yield was higher in those with CKDUA (48 of 281 patients) at 17.1%. The most common pathogenic variants were in PKD1, PKD2, COL4A3/4/5 and UMOD-associated tubulointerstitial disease.

Becherucci et al. [3]. published a study in 2023 which looked at 476 patients in 8 clinical categories (metabolic disorders, syndromic CKD, congenital abnormalities of the kidney and urinary tract [CAKUT], ciliopathies, tubulopathies, CKD unknown, collagenopathies and podocytopathies) with a suspected underlying genetic cause for CKD based on several different criteria (e.g. family history of kidney disease, resistance to treatments, extra renal involvement). They identified a genetic diagnosis in 70% of 156 adults. The suspected diagnosis was confirmed in 48% and modified in 19%.

Another study undertook WES on 114 families in Ireland with CKD [5] and identified a pathogenic mutation in 42 families (37%). Of the 42 positive results, in 17 families the clinical diagnosis was confirmed, in 9 there was a change to the clinical diagnosis, and in 16 a diagnosis was established for the first time. CKDUA was present in 34/114 families, and of these, 47% were found to have a pathogenic mutation, with diagnoses including cystic kidney disease, nephronophthisis, syndromic CAKUT, tubulointerstitial kidney disease, hypertensive renal disease and nephrocalcinosis/ nephrolithiasis.

Use of genetic testing has also been shown to be beneficial in the transplant setting with a study by Ottlewski et al. demonstrating that a gene panel involving 209 genes associated with ESKD was able to identify the cause for ESKD in 6/51 patients awaiting a transplant [4].

However, despite advances in genetic testing there remains a large proportion of CKDUA patients without a diagnosis. It is likely that as yet undiscovered environmental factors will play a role in some of these, along with vascular and subclinical hypertensive aetiologies. Furthermore, the largely non-specific biopsy findings identified in this study further complicate diagnosis in CKDUA.

Groundbreaking technologies such as RNA hybridization-based in situ sequencing are paving the way for more detailed examination of disease tissue at a single cell level [15]. Applying these to CKDUA biopsy tissues would identify dynamic alterations in various kidney cell types; allowing for analysis to determine how these interact with each other, the kidney parenchyma and immune cell types.

We were not able to perform genetic testing in our cohort, but there were only 4 patients within the CKDUA cohort who would have clearly met the criteria for genetic testing based on the literature (including age < 50 years, positive family history, presence of extra renal features and congenital/ cystic disease phenotypes) [2]. Given the high median age in our cohort (71.4 years), we postulate that it in fact represents a form of age-adjusted CKD. We support genetic testing in targeted cohorts with a high pre-test probability, but perhaps there is less of a role for it as an initial test in undifferentiated disease, particularly as outcomes were better than patients with DKD.

We identified the average rate of eGFR decline per year in the true CKDUA group and the H/D group. In normal ageing, at age 70 the median eGFR for men is 75 ml/min/1.73m^2^ and by age 80 this is 66 ml/min/1.73m^2^, equating to an average annual rate of eGFR loss of 0.9 ml/min/1.73m^2^ [16]. For women the median eGFR at age 70 is 72 ml/min/1.73m^2^, and by age 80 this is 63 ml/min/1.73m^2^, also equating to an average annual rate of eGFR loss of 0.9 ml/min/1.73m^2^ [16]. This suggests that the average rate of decline in our study is actually slightly better than healthy ageing, although this is likely reflected by the fact that some in our cohort were aged less than 70 (interquartile range 61.8–77.8 years).

**Fig. 2:**
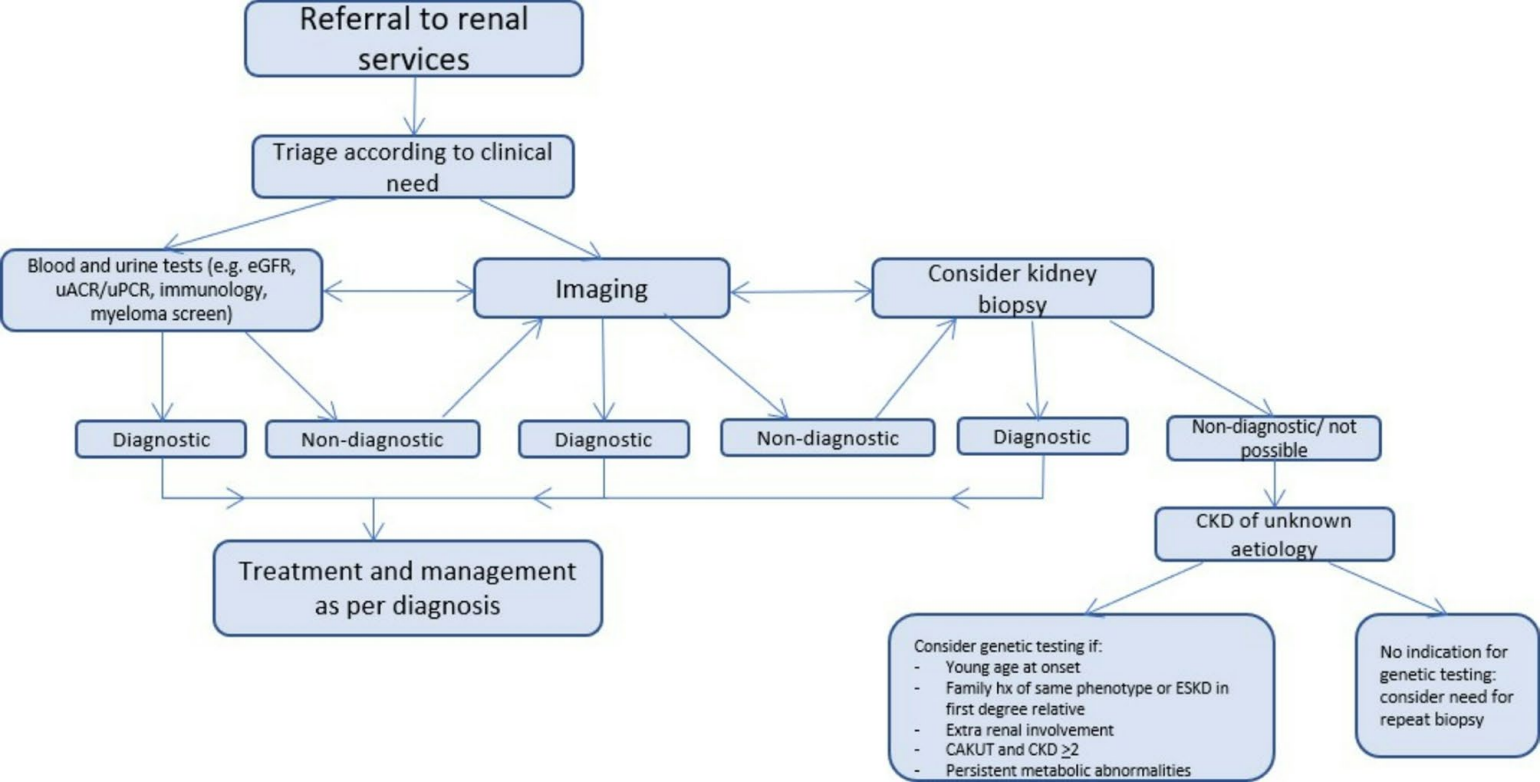
Proposed algorithm for use in clinical practice for patients referred into renal services with possible CKDUA

There are some limitations to the presented study. It was retrospective in nature involving patients recruited to SKS over a long time period (17 years). There were no specified criteria for diagnosing patients with CKDUA, although it is expected that conventional diagnostic workup was completed where applicable. One of the major limitations was that genetic testing was not performed in this cohort which would allow us to determine a diagnostic yield. Ideally if this study were to be repeated, we would recruit patients in a prospective manner who meet specific criteria for CKDUA, and have a high pre-test probability of an underlying genetic cause for their CKD based on previously published features. Diagnostic algorithms have previously been reported to aid clinicians when thinking about genetic testing in kidney disease [17]. Figure 2 depicts our own algorithm proposed for use when assessing patients referred into renal services who may go on to have CKDUA. We plan to adapt this algorithm further as we develop our own database of results and increase our experience of correlating phenotype with genotype, in conjunction with the NHS National Genomic Test Directory. During the last 20 months we have recruited patients into the SKS who are under 50 years of age. By incorporating family history, CKD stage and other metabolic parameters, we plan to determine those patients who are more likely to benefit from genetic testing.

There are several ways in which research into CKDUA may develop in the future. The optimal testing strategy for genetic testing is still being determined. Whole genome sequencing (WGS), as per the NHS National Genomic Test Directory, is reserved for those patients with steroid resistant nephrotic syndrome or select patients with an FSGS picture; cystic kidney disease; or the development of ESKD prior to age 36 [18]. By performing panel tests or WES for all other patients there remain large parts of the genome that are overlooked, and as such we may be missing a proportion of diagnoses. Performing WGS in the research setting would help us to answer this question. Furthermore, most of the work on genetic testing in kidney disease has been carried out on cohorts with a high pre-test probability of a genetic diagnosis. It is unclear if these results would extrapolate to the general CKD population and validation of these results in this setting would be beneficial. Finally, despite rapid advancements in our ability to test patients clinically for underlying genetic diagnoses, a large proportion of patients with CKDUA remain who are not diagnosed with a genetic condition. Further research on these patients, for example with biomarker analysis or newer technologies including RNA hybridization-based in situ sequencing, is needed to determine how to identify these patients early and to think about novel management strategies.

## Conclusions

Despite their being no specific treatment strategies for patients with CKDUA, in our real world study, these patients actually had improved outcomes (slower progression to RRT and a trend towards lower 5 year mortality) compared to the DKD cohort. Whilst we did not perform genetic testing in this study, the relatively increased age at presentation for this group (71.4 years) and low rates of associated family history of kidney disease (1%), suggests that blanket adoption of genetic testing is unlikely to be helpful. Further work is needed to determine usefulness of genetic testing in a real world cohort of patients with CKDUA.

## Supplementary Information

Below is the link to the electronic supplementary material.

Supplementary Material 1

## Data Availability

The datasets used and analysed during the current study are available from the corresponding author on reasonable request.

## Abbreviations

ADPKD: Autosomal dominant polycystic kidney disease
ADTKD: Autosomal dominant tubulointerstitial kidney disease
ACEi: Angiotensin-converting enzyme inhibitors
ARBs: Angiotensin receptor blockers
BMI: Body mass index
CAKUT: Congenital abnormality of the kidneys and urinary tract
CKD: Chronic kidney disease
CKDUA: Chronic kidney disease of unknown aetiology
COL4A: Collagen type 4 alpha 1 chain
DKD: Diabetic kidney disease
ESKD: End stage kidney disease
H/D CKD: Hypertensive/ diabetic chronic kidney disease
IQR: Interquartile range
MUC1: Mucin 1
RRT: Renal replacement therapy
SKS: Salford Kidney Study
UMOD: Uromodulin
WES: Whole exome sequencing
