# Supplementary material for: Chronic kidney disease of unknown aetiology: a real-world study
Source: BMC Nephrol. 2025 Oct 24;26:586. doi: 10.1186/s12882-025-04452-1 (PMC12551346; doi:10.1186/s12882-025-04452-1)
Supplement: Supplementary file 1 — Supplementary Material 1 [file 12882_2025_4452_MOESM1_ESM.docx]

### Supplemental material

Cox regression analysis was performed to determine if there were any associations between baseline variables and progression to either RRT or mortality in the CKDUA cohort (Supplemental Table 1). In univariate analysis the following variables were associated with increased risk for developing RRT: higher uPCR and phosphate; and the following variables were protective in relation to RRT risk: age, White ethnicity, higher eGFR, higher haemoglobin and higher albumin. In multivariate analysis only age (HR 0.96, CI 0.94-0.98, p<0.001) and eGFR (HR 0.87, CI 0.83-0.92, p<0.001) had statistically significant effects on risk of progression to RRT (both protective).

When looking at risk of mortality, the following factors were associated with increased risk: increasing age, hypertension, congestive cardiac failure, peripheral vascular disease, higher phosphate and higher uPCR; and the following were protective: higher eGFR, albumin and haemoglobin. In the multivariate analysis only increasing age (HR 1.05, CI 1.04-1.07, p<0.001), higher uPCR (HR 1.003, CI 1.002-1.004, p<0.001) and higher haemoglobin (HR 0.98, CI 0.97-0.99, p<0.001) remained statistically significant.

**Supplemental Table 1. Univariate and multivariate cox regression analysis assessing factors associated with RRT and mortality.**

|  | Factors associated with RRT | | | | Factors associated with mortality | | | |
| --- | --- | --- | --- | --- | --- | --- | --- | --- |
|  | Univariate analysis | | Multivariate analysis | | Univariate analysis | | Multivariate analysis | |
| Variable | Hazard ratio (95% CI) | P value | Hazard ratio (95% CI) | P value | Hazard ratio (95% CI) | P value | Hazard ratio (95% CI) | P value |
| Age | 0.97 (0.95-  0.98) | **<0.001** | 0.96 (0.94-  0.98) | **<0.001** | 1.06 (1.04-  1.07) | **<0.001** | 1.05 (1.04-  1.07) | **<0.001** |
| Male gender | 1.52 (0.86-  2.69) | 0.152 | - | - | 1.06 (0.81-  1.39) | 0.675 | - | - |
| White  ethnicity | 0.26 (0.117-  0.58) | **0.001** | 0.81 (0.26-  2.50) | 0.709 | 2.41 (0.78-  7.67) | 0.123 | - | - |
| Smoker | 1.15 (0.67-  1.97) | 0.622 | - | - | 1.30 (0.99-  1.71) | 0.064 | - | - |
| Alcohol intake | 1.68 (0.99-  2.84) | 0.053 | - | - | 0.99 (0.75-  1.29) | 0.918 | - | - |
| BMI | 0.95 (0.90-  1.01) | 0.076 | - | - | 0.99 (0.96-  1.01) | 0.264 | - | - |
| Hypertension | 0.93 (0.44-  1.97) | 0.930 | - | - | 1.85 (1.14-  3.00) | **0.012** | 1.46 (0.86-  2.50) | 0.162 |
| Diabetes | 0.45 (0.163-  1.25) | 0.126 | - | - | 1.36 (0.97-  1.91) | 0.078 | - | - |
| Angina | 0.53 (0.23-  1.24) | 0.143 | - | - | 1.34 (0.98-  1.84) | 0.070 | - | - |
| MI | 0.407 (0.13-  1.30) | 0.130 | - | - | 1.552 (1.10-  2.19) | 0.012 | - | - |
| CCF | 1.24 (0.62-  2.47) | 0.538 | - | - | 1.92 (1.40-  2.64) | **<0.001** | 1.31 (0.92-  1.86) | 0.139 |
| CVA | 1.31 (0.47-  3.62) | 0.607 | - | - | 1.58 (0.97-  2.56) | 0.064 | - | - |
| PVD | 1.20 (0.51-  2.81) | 0.677 | - | - | 2.04 (1.42-  2.92) | **<0.001** | 1.34 (0.91-  1.98) | 0.140 |
| ACEi/ARB | 0.98 (0.58-  1.68) | 0.949 | - | - | 1.15 (0.88-  1.51) | 0.302 | - | - |
| Statin | 0.69 (0.40-  1.18) | 0.174 | - | - | 1.16 (0.89-  1.52) | 0.279 | - | - |
| eGFR | 0.90 (0.87-  0.93) | **<0.001** | 0.87 (0.83-  0.92) | **<0.001** | 0.98 (0.97-  0.99) | **<0.001** | 1.007 (0.995-  1.02) | 0.233 |
| Albumin | 0.90 (0.83-  0.97) | **0.003** | 0.93 (0.83-  1.04) | 0.191 | 0.93 (0.89-  0.96) | **<0.001** | 0.97 (0.93-  1.02) | 0.215 |
| Haemoglobin | 0.97 (0.96-  0.99) | **0.001** | 0.98 (0.96-  1.01) | 0.138 | 0.975 (0.97-  0.98) | **<0.001** | 0.98 (0.97-  0.99) | **<0.001** |
| Phosphate | 13.7 (5.95-  31.3) | **<0.001** | 1.75 (0.55-  5.58) | 0.345 | 2.83 (1.59-  5.06) | **<0.001** | 1.68 (0.80-  3.53) | 0.172 |
| uPCR | 1.003 (1.002-  1.005) | **<0.001** | 1.001 (1.00-  1.004) | 0.374 | 1.002  (1.000-  1.003) | **0.009** | 1.003 (1.002-  1.004) | **<0.001** |
| Discrepancy in kidney size on  USS | 1.07 (0.24-  4.69) | 0.931 | - | - | 1.05 (0.43-  2.58) | 0.916 | - | - |
| Cysts on USS | 1.29 (0.71-  2.36) | 0.401 | - | - | 1.28 (0.92-  1.78) | 0.144 | - | - |
| CTD screen  abnormality | 0.70 (0.34-  1.43) | 0.326 | - | - | 0.75 (0.53-  1.06) | 0.106 | - | - |

ACEi, angiotensin converting enzyme inhibitor; ARB, angiotensin receptor blocker; BMI, body mass index; CCF, congestive cardiac failure; CTD, connective tissue disease; CVA, cerebro-vascular accident; eGFR, estimated glomerular filtration rate; MI, myocardial infarction; PVD, peripheral vascular disease; RRT, renal replacement therapy; uPCR, urine protein creatinine ratio; USS, ultrasound scan.
